# Insufficient maternal iodine intake is associated with subfecundity, reduced foetal growth, and adverse pregnancy outcomes in the Norwegian Mother, Father and Child Cohort Study

**DOI:** 10.1186/s12916-020-01676-w

**Published:** 2020-08-11

**Authors:** Marianne Hope Abel, Ida Henriette Caspersen, Verena Sengpiel, Bo Jacobsson, Helle Margrete Meltzer, Per Magnus, Jan Alexander, Anne Lise Brantsæter

**Affiliations:** 1 Department of Chronic Diseases and Ageing, Division of Mental and Physical Health, Norwegian Institute of Public Health, P.O. Box 222, Skøyen, NO-0213 Oslo, Norway; 2 Department of Environmental Health, Division of Infection Control, Environment and Health, Norwegian Institute of Public Health, P.O. Box 222, Skøyen, NO-0213 Oslo, Norway; 3 Department of Obstetrics and Gynaecology, Sahlgrenska University Hospital, SE 416 85 Gothenburg, Sweden; 4 Department of Obstetrics and Gynaecology, Institute of Clinical Sciences, University of Gothenburg, SE 416 85 Gothenburg, Sweden; 5 Department of Genetics and Bioinformatics, Division of Health Data and Digitalisation, Institute of Public Health, P.O. Box 222, Skøyen, NO-0213 Oslo, Norway; 6 Division of Infection Control, Environment and Health, Norwegian Institute of Public Health, P.O. Box 222, Skøyen, NO-0213 Oslo, Norway; 7 Centre for Fertility and Health, Norwegian Institute of Public Health, P.O. Box 222, Skøyen, NO-0213 Oslo, Norway

**Keywords:** Mild-to-moderate iodine deficiency, Iodine intake, Iodine supplement, Pregnancy cohort, Foetal growth, Preeclampsia, Preterm delivery, Subfecundity, The Norwegian Mother, Father and Child Cohort Study (MoBa)

## Abstract

**Background:**

Severe iodine deficiency impacts fertility and reproductive outcomes. The potential effects of mild-to-moderate iodine deficiency are not well known. The aim of this study was to examine whether iodine intake was associated with subfecundity (i.e. > 12 months trying to get pregnant), foetal growth, and adverse pregnancy outcomes in a mild-to-moderately iodine-deficient population.

**Methods:**

We used the Norwegian Mother, Father and Child Cohort Study (MoBa) and included 78,318 pregnancies with data on iodine intake and pregnancy outcomes. Iodine intake was calculated using an extensive food frequency questionnaire in mid-pregnancy. In addition, urinary iodine concentration was available in a subsample of 2795 pregnancies. Associations were modelled continuously by multivariable regression controlling for a range of confounding factors.

**Results:**

The median iodine intake from food was 121 μg/day and the median urinary iodine was 69 μg/L, confirming mild-to-moderate iodine deficiency. In non-users of iodine supplements (*n* = 49,187), low iodine intake (< 100–150 μg/day) was associated with increased risk of preeclampsia (aOR = 1.14 (95% CI 1.08, 1.22) at 75 vs. 100 μg/day, *p* overall < 0.001), preterm delivery before gestational week 37 (aOR = 1.10 (1.04, 1.16) at 75 vs. 100 μg/day, *p* overall = 0.003), and reduced foetal growth (− 0.08 SD (− 0.10, − 0.06) difference in birth weight *z*-score at 75 vs. 150 μg/day, *p* overall < 0.001), but not with early preterm delivery or intrauterine death. In planned pregnancies (*n* = 56,416), having an iodine intake lower than ~ 100 μg/day was associated with increased prevalence of subfecundity (aOR = 1.05 (1.01, 1.09) at 75 μg/day vs. 100 μg/day, *p* overall = 0.005). Long-term iodine supplement use (initiated before pregnancy) was associated with increased foetal growth (+ 0.05 SD (0.03, 0.07) on birth weight *z*-score, *p* < 0.001) and reduced risk of preeclampsia (aOR 0.85 (0.74, 0.98), *p* = 0.022), but not with the other adverse pregnancy outcomes. Urinary iodine concentration was not associated with any of the dichotomous outcomes, but positively associated with foetal growth (*n* = 2795, *p* overall = 0.017).

**Conclusions:**

This study shows that a low iodine intake was associated with restricted foetal growth and a higher prevalence of preeclampsia in these mild-to-moderately iodine-deficient women. Results also indicated increased risk of subfecundity and preterm delivery. Initiating iodine supplement use in pregnancy may be too late.

## Background

Iodine is an essential micronutrient and an integral part of the thyroid hormones triiodothyronine (T3) and thyroxine (T4). The thyroid hormones regulate multiple metabolic processes that are important in growth, metabolism, and reproduction. Thyroid dysfunction has been linked to menstrual disturbances, reduced fecundity (i.e. ability to become pregnant), miscarriage, gestation-induced hypertension, preterm delivery, and reduced foetal growth [1].

Iodine deficiency is highly prevalent in both low- and high-income countries, even though deficiency is easily preventable through salt iodization strategies as recommended by the World Health Organization (WHO) [2]. Worldwide, iodine nutrition is recognized as one of the key determinants of thyroid dysfunction [3]. Recent findings in two population-based cohort studies have indicated that even mild-to-moderate iodine deficiency may affect thyroid function in pregnant women [4, 5]. In addition, an abrupt increase in iodine intake caused by introduction of salt iodization programmes or iodine supplement use might temporarily affect thyroid function in populations that are mild-to-moderately iodine deficient [4, 6]. While it is well documented that severe iodine deficiency poses reproductive risks, including abortions, stillbirths, and impaired neurodevelopment, the potential impact of mild-to-moderate iodine deficiency on fertility and pregnancy outcomes remains largely unknown [7].

It has been well known for many decades that iodine deficiency reduces fecundity in livestock [8], but we have identified only one study that has investigated this association in humans [9]. The study sample included 467 women trying to get pregnant, and a low urinary iodine concentration (UIC < 50 μg/g creatinine) was significantly associated with delayed conception compared to a UIC within the normal range (≥ 100 μg/g creatinine) [9]. A few studies have investigated associations between iodine status and risk of adverse pregnancy outcomes (i.e. preeclampsia, preterm delivery, pregnancy loss) and/or birth anthropometrics in mild-to-moderate iodine deficiency [10–14], but the studies were underpowered to identify potential minor changes in risks for dichotomous outcomes, and most have reported null findings. For birth weight, some studies have reported reduced birth weight in mild-to-moderate iodine deficiency [13, 14], but a recent systematic review found no evidence of the effect of iodine supplement or salt iodization on prenatal growth in mild-to-moderate iodine deficiency [15]. However, the quality of the evidence was assessed as very low [15]. Therefore, there is still a major knowledge gap as to whether mild-to-moderate iodine deficiency affects fertility and pregnancy outcomes.

In the present study, we used data from the Norwegian Mother, Father and Child Cohort Study (MoBa), a large pregnancy cohort with detailed information about food intake, supplement use, and a number of obstetric outcomes [16]. We have previously documented that the MoBa pregnant women were mild-to-moderately iodine deficient at a group level defined by WHO criteria [4] and that there was a large variation in iodine intake between participants due to few food sources (mainly milk and fish) and supplement use [17]. We also found that iodine intake was associated with thyroid function in pregnancy and that a low maternal iodine intake in pregnancy was associated with poorer child neurocognitive development at ages 3 and 8 years [4, 18, 19]. In MoBa, three different exposures are available as measures of iodine intake: calculated iodine intake from food, reported use of iodine-containing supplements, and UIC in a subsample of women. Consequently, this large prospective study offered a unique opportunity to add new knowledge about the role of mild-to-moderate iodine deficiency on subfecundity and adverse pregnancy outcomes.

## Methods

The aim of the current study was to examine if iodine intake was associated with subfecundity (i.e. > 12 months trying to get pregnant), stillbirth, preeclampsia, preterm delivery, and birth anthropometrics in a large cohort of mild-to-moderately iodine-deficient women.

### Subjects and design

**Fig. 1:**
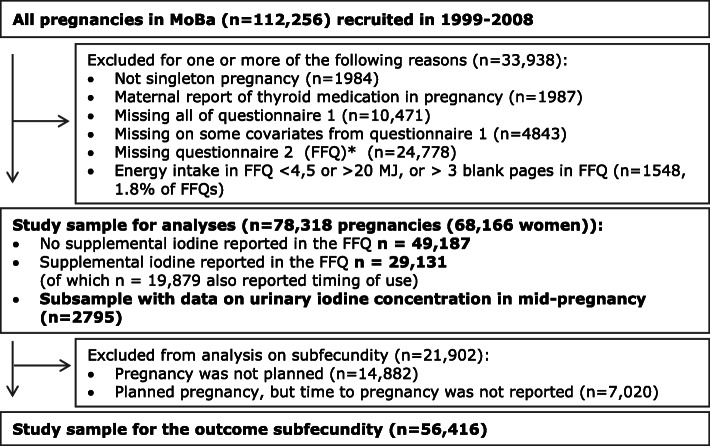
Flow chart of inclusion. Only complete cases were included (5% had missing values on one or more covariates). Asterisk indicates that the food frequency questionnaire (FFQ) was in use from 2002

This study is based on MoBa, a prospective population-based pregnancy cohort initiated and maintained by the Norwegian Institute of Public Health [16]. Women pregnant in their first trimester were recruited from all over Norway during years 1999 to 2008 and were asked to answer questionnaires (available in Norwegian only) at regular intervals during pregnancy and after birth. Pregnancy and birth records from the Medical Birth Registry of Norway (MBRN) are linked to the MoBa database [20]. The women consented to participation in 41% of the pregnancies. The cohort now includes 114,500 children, 95,200 mothers, and 75,200 fathers. The current study is based on version 10 of the quality-assured data files released for research in 2017 and restricted to participants recruited from 2002 to 2008 because the MoBa food frequency questionnaire (FFQ) was included in the data collection from March 2002. To be included in the current study, participants had to have responded to (i) a baseline questionnaire (Q1) around gestational week (GW) 17 covering general health and sociodemographic information and (ii) the FFQ (Q2) around GW 22 and (iii) to be registered in MBRN with a singleton delivery. We excluded women who reported use of thyroid medication at any time during pregnancy. Given the large sample size and low rates of missing values (≤ 5%), only pregnancies with information on all covariates were included. FFQs with more than three blank pages or with calculated energy intakes outside the range 4.5–20 MJ/day were excluded [21]. Some women participate in MoBa with more than one pregnancy. The final study population comprised 78,318 pregnancies (68,166 women) for the analyses of pregnancy outcomes, and 56,416 planned pregnancies for the analysis of subfecundity. UIC was available in a subsample of 2795 pregnancies and was measured in GW 18. A flow chart of inclusion is illustrated in Fig. 1.

### Exposure variable: iodine intake

The MoBa FFQ is a comprehensive semi-quantitative questionnaire specifically designed and validated for MoBa [21, 22]. Participants responded to the FFQ around GW 22 and were asked to report their average intake since becoming pregnant (GW 0–22). Food frequencies were converted to food amounts using standard Norwegian portion sizes, and daily intakes of energy and nutrients were calculated using FoodCalc [23] and the Norwegian food composition table [24]. Data on the content of more than 1000 food supplements was collected from suppliers [25]. Participants with unrealistic energy intakes in the FFQs (i.e. < 4.5 or > 20 MJ/day) or more than three blank pages were excluded from the study sample. Iodine intake measured by the FFQ has previously been validated, and it shows good agreement with 4 days weighed food diary [22] and urinary iodine concentration (UIC) [4, 22]. Use of supplements was reported in the FFQ and in the general questionnaires for different time periods. Timing of the first reported use was coded in four categories (never, weeks 0–26 before pregnancy, GW 0–12 and GW > 12).

Although we assessed iodine intake in pregnancy with an FFQ covering the average food intake in the first 4–5 months of pregnancy, we propose that this iodine intake also can serve as an indicator of habitual iodine intake prior to pregnancy.

Urine samples were collected at the routine ultrasound examination offered free of charge to all Norwegian women in GW 18. UIC was determined at the National Institute for Health and Welfare in Helsinki, Finland, by inductively coupled plasma–mass spectrometry using an Agilent 7800 ICP-MS system (Agilent Technologies Inc., Santa Clara, CA, USA). The limit of quantification was 2 μg/L, and the linearity was excellent up to 1500 μg/L (*r* = 0.9999). The coefficient of variation was 2–3%.

### Pregnancy and birth outcomes

All outcomes except subfecundity were based on information in the MBRN. The subfecundity outcome, which applied only to planned pregnancies, was based on reported time (months) to conception reported in the general questionnaire in GW 17.

*Subfecundity* was defined as > 12 months trying to get pregnant for planned pregnancies (72% of the women had reported that pregnancy was planned and also reported time to pregnancy). The wording of the question was “How many months did you have regular intercourse without contraception before you became pregnant?” Women with in vitro fertilization were not excluded.

*Intrauterine death* was defined as death before birth (0.26%) or death during birth (0.02%). It also included registered intrauterine deaths where the time of death was not specified (0.06%). Abortions of live foetuses were not included.

*Preeclampsia* was defined if any of the following conditions were checked off in the pregnancy record: (i) HELLP syndrome (i.e. haemolysis, elevated liver enzymes, and low platelet count), (ii) eclampsia, (iii) early-onset preeclampsia (diagnosed before 34 weeks), (iv) mild preeclampsia, or (v) severe preeclampsia. In Norway, all pregnant women receive free antenatal care. Blood pressure measurement and proteinuria analysis are carried out at each antenatal visit. According to guidelines issued by the Norwegian Society of Obstetrics and Gynaecology, the diagnostic criteria for preeclampsia are blood pressure > 140/90 after 20 weeks gestation, combined with proteinuria greater than + 1 dipstick on at least two occasions.

*Preterm delivery* was defined as delivery before GW 37 + 0 and as early preterm when delivered before GW 32 + 0. Gestational age in days was determined based on the routine ultrasound examination given free of charge to all women in GW 18, or it was calculated based on time from the first day of the last menstruation period in the few women where ultrasound data was missing (1.9%). Preterm delivery was also categorized by delivery initiation, i.e., spontaneous preterm delivery (preterm labour or preterm prelabour rupture of the membranes) or iatrogenic preterm delivery (induced or primary caesarean delivery on maternal or foetal indications).

*Birth weight* was examined as four outcomes: crude birth weight (gram); standardized birth weight (*z*-score), i.e. birth weight adjusted for child sex and gestational age based on all deliveries in MBRN; small for gestational age (SGA, gestational age- and sex-adjusted *z*-score < 10 percentile); and large for gestational age (LGA, gestational age- and sex-adjusted *z*-score > 90 percentile). Outcomes on birth weight and gestational age at birth were recoded to missing if birth weight for gestational age and sex was > ± 5 standard deviations from the mean (*n* = 13) since these data suggested misreporting of either birth weight or gestational length.

*Head circumference* was examined as a crude measure (cm). Recorded head circumference > 43 cm (0.05%) was suspected as misreporting and recoded to missing.

*Placenta weight* was examined as a crude measure (gram). Recorded placenta weight > 3000 g (0.1%) was suspected as misreporting and recoded to missing.

### Other variables

Covariates were included in the statistical models based on previous knowledge and directed acyclic graphs (DAGs, see Additional file 1: Figure S1). Maternal age at the time of birth was obtained from the birth registry. Maternal pre-pregnancy body mass index (BMI), education (≤ 12, 13–16, ≥ 17 years), marital status (married/cohabitant: yes/no), parity (previous pregnancies ≥ 22 weeks: 0, 1, ≥ 2), history of chronic illness (asthma, diabetes, inflammatory bowel disease, rheumatic disease, epilepsy, multiple sclerosis, or cancer before or during pregnancy: yes/no), smoking before pregnancy (no, occasional, daily), use of in vitro fertilization in current pregnancy (yes/no), and use of a folic acid supplement within the interval from 4 weeks before to 8 weeks after conception (yes/no) were obtained from questionnaire 1 (GW 17). Maternal energy intake, fibre intake (as marker of a healthy diet), use of probiotic milk products (yes/no), and total intake of the omega-3 fatty acids EPA and DHA were calculated based on the FFQ (GW 22). Also, use of dietary supplements other than the ones commonly recommended for pregnant women (i.e. other than vitamin D, folic acid, and iron) was obtained from the FFQ (yes/no). Information on smoking in pregnancy was obtained from questionnaire 1 and, if available, questionnaires 3 (GW 30) and 4 (child’s age 6 months) (three categories: no reported smoking in pregnancy, reported occasional smoking or stopped smoking before GW 12, and daily smoking at any time in pregnancy and had not stopped smoking before GW 12).

### Statistical methods

Statistical analyses were performed in STATA (version 15.0; Stata Corp., College Station, TX). Associations were estimated by linear regression analyses for continuous outcomes and logistic regression for dichotomous outcomes. In sensitivity analyses for the outcome subfecundity, Cox regression was used to model time to pregnancy as a continuous variable.

Associations between iodine from food and outcomes, and UIC and outcomes, were modelled flexibly with restricted cubic splines. Since some mothers were included with more than one pregnancy (14%), we specified person clusters by using the option vce (cluster person_ID) in models in STATA, which relaxes the assumption of independence of the observations and produces robust estimates of variance. *p* values are reported for overall associations between continuous exposures and outcomes (testing H0: no association) by testing the coefficients of all spline transformations equal to zero. In addition, tests for non-linearity were performed by testing the coefficients of the second and third spline transformations equal to zero. Covariates were included in the models based on DAGs. Continuous covariates (e.g. maternal age, BMI, and energy intake) were modelled flexibly by restricted cubic splines if there was evidence of non-linear associations (determined by inspecting the estimated associations while controlling for other covariates in the model); otherwise, they were modelled linearly. We report the specific covariates for each outcome in the respective tables and figures. Tabular results of the graphs included in this paper are provided in Additional file 1: Tables S1-S3.

Iodine supplement use was modelled as (1) any reported iodine supplement use in GW 0–22 and (2) timing of first reported use (never, started before conception, started in GW 0–12, started in GW 13–22). Potential effect modification by iodine intake from food was explored including an interaction term between iodine from food (modelled by restricted cubic splines) and the supplement use variable. Potential interactions were explored by testing all interaction coefficients equal to zero. If the interaction terms were not statistically significant, iodine from food was not included in the final models. Women in the control group were all non-users of iodine-containing supplements. In the sensitivity analysis, we restricted the control group to women who had reported use of dietary supplements other than the standard, recommended ones. The use of this restricted control group could control for the behaviour of taking an extra vitamin/mineral supplement and could to some extent also control for other nutrients in the multisupplements.

We did not include power calculations as no relevant effect estimates were available in comparable populations.

A *p* value < 0.05 was considered statistically significant, and results are reported including robust 95% confidence intervals (CI). Only participants with complete data on all covariates were included in the analyses due to the low rate of missing values (in total, 5% of eligible participants had missing on one or more covariates).

## Results

The median calculated iodine intake from food was 121 μg/day (IQR 89, 161 μg/day) (Table 1). Seventy-four per cent had an iodine intake from food lower than the estimated average requirement for pregnant women defined by the Institute of Medicine (i.e. < 160 μg/day) [26], and only 4.6% reached the recommended intake in pregnancy by the WHO (i.e. ≥ 250 μg/day) [27] without including supplements. The median UIC (measured in *n* = 2795) was 69 μg/L and 37% had UIC < 50 μg/L. This is well below the WHO recommendation (i.e. median UIC ≥ 150 μg/L for pregnant women and median ≥ 100 μg/L for non-pregnant) [27].

**Table 1:** Iodine exposures by characteristics of the study population (*n* = 78,318)

	Study population	Iodine from food, median (IQR), μg/day	Iodine supplement, GW 0–22, %	UIC^a^ (*n* = 2795), median (IQR), μg/L
Study sample, *n* (%)	78,318 (100)	121 (89, 161)	37	69 (35, 116)
Maternal age at delivery, mean (SD), years	30.2 (4.5)			
< 25	11	122 (85, 172)	35	68 (38, 108)
25–34	72	121 (89, 160)	37	67 (35, 115)
≥ 35	17	122 (91, 160)	38	73 (37, 120)
Pre-pregnancy BMI, mean (SD), kg/m^2^	24.0 (4.3)			
< 18.5	3.0	123 (89, 163)	40	64 (31, 128)
18.5–24.9	66	122 (90, 161)	38	67 (34, 114)
25–30	22	120 (87, 163)	36	69 (34, 118)
> 30	9.5	118 (84, 161)	35	75 (44, 119)
Parity, %				
0	47	119 (87, 160)	42	68 (35, 116)
1	36	122 (90, 161)	34	69 (34, 118)
2 or more	17	127 (94, 168)	30	69 (40, 104)
Maternal education, %				
≤ 12 years	31	122 (86, 168)	33	70 (39, 115)
13–16 years	43	122 (89, 161)	38	68 (34, 114)
> 16 years	27	120 (90, 155)	40	68 (34, 120)
Married/cohabitant, %				
Yes	96.7	121 (89, 161)	37	69 (35, 116)
No	3.3	123 (88, 169)	37	69 (30, 103)
Smoking in pregnancy, %				
No	79	122 (89, 161)	38	70 (37, 119)
Occasionally	16	120 (87, 162)	37	61 (31, 106)
Daily	5.0	123 (87, 172)	32	62 (32, 95)
Chronic illness, %				
No	90	122 (90, 162)	37	68 (35, 116)
Yes	10	116 (83, 158)	41	72 (34, 118)
Couples income				
Low	26	125 (90, 169)	35	72 (39, 115)
Medium	41	122 (90, 163)	37	66 (34, 118)
High	30	117 (87, 153)	40	69 (34, 114)
Missing	2.7	129 (92, 175)	34	71 (47, 104)
Iodine supplement in pregnancy, %				
No	63	122 (89, 162)	0	59 (32, 100)
Yes	37	121 (89, 161)	100	85 (45, 140)
Reported use in GW 17–20	17	122 (89, 161)	100	99 (54, 154)
Vitamin D supplement (%)	77	123 (91, 162)	47	70 (36, 120)
Multivitamin/multimineral (%)	49	122 (90, 162)	66	76 (39, 129)
Folic acid before/early pregnancy (%)	73	121 (89, 159)	42	70 (36, 120)
Maternal energy intake, median (IQR), MJ	9.4 (7.9, 11.1)			
Iodine from food, median (IQR), μg/day	121 (89, 161)			
< 75	16	61 (50, 68)	38	51 (27, 96)^b^
75–149.9	54	112 (94, 129)	37	68 (34, 114)^b^
≥ 150	31	187 (166, 223)	37	78 (43, 129)^b^

^a^Urinary iodine concentration (UIC) was measured in a subsample of *n* = 2795 pregnant women in mean gestational week 18.5 (SD 1.3). Iodine intake from food and use of iodine-containing supplements were comparable in this subgroup versus the whole study sample

^b^Restricted to non-users of iodine-containing supplements

Some groups of women could be identified as having a particularly low UIC, for example all non-users of iodine-containing supplements (63% of all participants, median UIC 59 μg/L). Furthermore, non-iodine supplement users who consumed less than 3 dL milk/yoghurt per day (25% of all participants) had a median UIC of 48 μg/L, and those who excluded dairy products entirely from their diet (1.5% of all participants) had a median UIC of 32 μg/L. Iodine intake from food correlated strongly with the reported intake of milk/yoghurt (Spearman *r* = 0.85) and moderately with the intake of lean fish (Spearman *r* = 0.32). The women with available UIC measurements (3.6% of the total study population) had a similar calculated iodine intake from food by the FFQ, and equal frequency distribution of reported iodine supplement use as the women without UIC measurements.

There were marginal variations in iodine intake from food, use of iodine-containing supplements, and UIC by background characteristics (Table 1). Table S4 in Additional file 1 shows background characteristics by categories of iodine intake from food and use of iodine-containing supplements. Iodine from food was weakly correlated with fibre intake (Spearman *r* = − 0.08 after adjusting for energy intake) indicating a weak negative association with this indicator of a healthy diet.

Participants in MoBa that were excluded from the study sample due to missing values on one or more of the covariates (*n* = 4305, 5%) did not differ in iodine intake from food, UIC, or any of the outcomes (prevalence of subfecundity, intrauterine death, preeclampsia, preterm delivery, or birth weight *z*-score). The prevalence of iodine supplement use was lower in excluded participants (34% vs. 37%, *p* < 0.001).

Descriptive statistics of the pregnancy and birth outcome variables is provided in Table 2. There were some overlaps between the outcomes. Of the preeclamptic pregnancies (3.8%), 23% were preterm deliveries and 24% resulted in SGA infants, while 9.4% of preeclamptic pregnancies were both preterm and SGA.

**Table 2:** Pregnancy and birth outcomes (*n* = 78,318)

	**Study sample**^a^	**Median (IQR)**	**90% range**
Time to pregnancy (months)^b^	56,416	1.5 (0.5, 6)	0.5–14
Gestational length (weeks)	77,995	40 (39, 41)	37–42
Birth weight (g)	78,210	3610 (3275, 3945)	2704–4460
Birth weight (*z*-score by gestational age and sex)	77,949	0.08 (− 0.54, 0.73)	− 1.42–1.77
Placenta weight (g)	76,343	660 (580, 760)	450–940
Head circumference (cm)	76,693	35 (34, 36)	33–38
	**Study sample**^a^	**Number with outcome**	**Percent with outcome**
Subfecundity (> 12 months) ^b^	56,416	6078	10.8 (10.0^c^)
Intrauterine death	78,318	270	0.34 (0^c^)
Preeclampsia	78,318	2936	3.8 (2.7^c^)
Preterm delivery (< GW 37)	77,995	3889	5.0 (2.9^c^)
Spontaneous preterm delivery (< 37 weeks)	77,940	2211	2.8 (2.2^c^)
Early preterm delivery (< 32 weeks)	77,995	612	0.78 (0.04^c^)
Small for gestational age (< 10 percentile)	77,949	7637	9.8 (8.2^c^)
Large for gestational age (> 90 percentile)	77,949	7539	9.7 (10.6^c^)

^a^Small differences in numbers are explained by missing data

^b^Only for planned pregnancies with available data on time to pregnancy (72%)

^c^Prevalence in the subsample with UIC measurements (*n* = 2795)

### Subfecundity

**Fig. 2:**
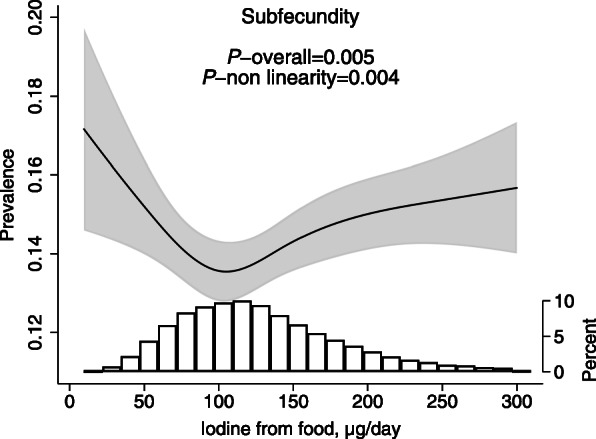
Habitual iodine intake from food (GW 0–22) and estimated prevalence of subfecundity (> 12 months trying to get pregnant) in planned pregnancies (*n* = 56,416, 10.8% subfecundity). The association was modelled by logistic regression adjusting for maternal age, BMI, parity, education, smoking before pregnancy, energy intake, and fibre intake. The curve represents the estimated prevalence when all covariates are set to their means, and the shaded area illustrates the 95% robust confidence interval. The histogram shows the distribution of the exposure. For the crude association, see Additional file 1: Figure S2

The association between iodine intake from food and subfecundity was U-shaped, and iodine intake in the interval between approximately 100 and 150 μg/day was associated with the lowest likelihood of subfecundity (Fig. 2). Compared with an intake of 100 μg/day (reference, OR = 1), the aOR at 75 μg/day was 1.05 (95% CI 1.01, 1.09), and at 50 μg/day, aOR was 1.14 (95% CI 1.04, 1.26), *p* overall = 0.005. There was no data in MoBa on supplement use before 6 months pre-pregnancy; thus, supplement use was not included as a variable in the model for subfecundity, and women were included in the analysis regardless of their reported iodine supplement use later. However, in sensitivity analyses, women who reported use of iodine-containing supplements in the time period 26–9 weeks before conception (7.8%) were excluded, and this did not change the results (results not shown). Time to pregnancy in months was also modelled as a continuous variable by Cox regression, and the findings were consistent with the results for subfecundity. In the subsample of women with UIC measurements (GW 18) who did not report current supplement use at the time of UIC sampling, there was no association between UIC in pregnancy and prevalence of subfecundity (*n* = 1260, *p* = 0.40).

### Intrauterine death, preeclampsia, and preterm delivery

**Fig. 3:**
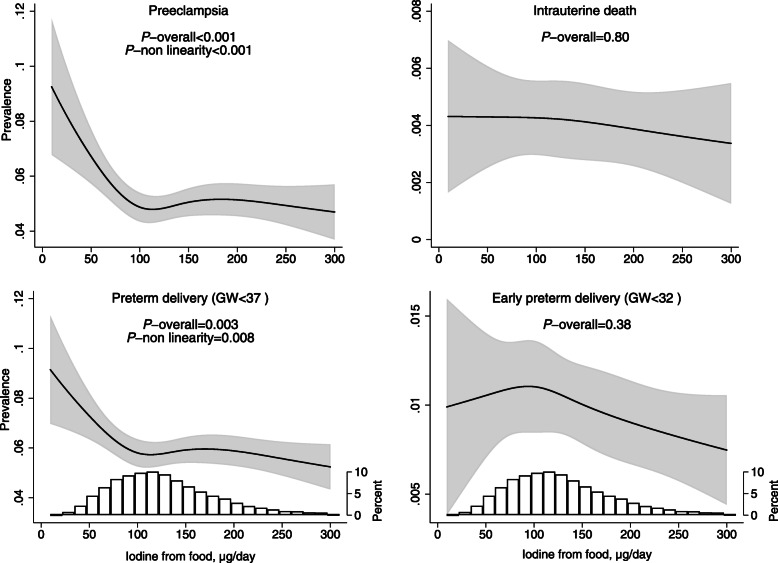
Iodine from food and adverse pregnancy outcomes in non-users of iodine-containing supplements. Sample size: intrauterine death *n* = 49,187 (0.35% intrauterine deaths), preeclampsia *n* = 49,187 (3.8% preeclampsia), and preterm delivery *n* = 48,981 (5.0% preterm and 0.84% early preterm). The associations were modelled by logistic regression adjusting for maternal age, BMI, parity, education, smoking in pregnancy, energy intake, and fibre intake. For the crude associations, see Additional file 1: Figure S3

In non-users of iodine-containing supplements, iodine intake from food lower than ~ 100 μg/day was associated with increased prevalence of preeclampsia and preterm delivery, but not with early preterm delivery or intrauterine death (Fig. 3). Compared to an intake of 100 μg/day (reference), an intake of 75 μg/day was associated with an increased risk of preeclampsia (aOR = 1.14% (95% CI 1.08, 1.22)) and an increased risk of preterm delivery (aOR = 1.10 (95% CI 1.04, 1.16)). At an intake of 50 μg/day, the adjusted odds ratio of preeclampsia was 1.41 (95% CI 1.20, 1.64) and for preterm delivery, it was 1.28 (95% CI 1.11, 1.47) (Fig. 3).

For preterm delivery (less than week 37), excluding participants with preeclampsia did not change the results (results not shown). When subdividing into spontaneous and iatrogenic preterm delivery, the shape of the associations looked similar, but only remained significant for iatrogenic PTD (*p* < 0.001) (Additional file 1: Figure S4). We did not examine associations between UIC and intrauterine death (prevalence 0%) or early preterm delivery (prevalence 0.04%) due to lack of power to detect potential differences in the subsample of women with available UIC data. We found no associations between UIC and preterm delivery or preeclampsia (Additional file 1: Figure S5).

Use of an iodine-containing supplement in GW 0–20 was borderline associated with a reduced risk of intrauterine death; however, the association was attenuated when restricting the reference group (i.e. non-use of iodine-containing supplements) to only include women who reported use of dietary supplements other than the ones commonly recommended for pregnant women (Table 3). For preeclampsia, long-term supplement use (i.e. use initiated before pregnancy) was associated with a decreased prevalence (aOR = 0.85 (95% CI 0.74, 0.98), *p* = 0.022), and the effect estimate was not attenuated when restricting the reference group. Regarding preterm delivery, the results were not consistent. Long-term supplement use was associated with an increased risk of preterm delivery, also when restricting to spontaneous preterm delivery, whereas more short-term supplement use (initiated in pregnancy) was associated with a decreased risk of early preterm delivery.

**Table 3:** Use of iodine-containing supplements and pregnancy and birth outcomes

	Number	Crude models		Adjusted models^**a**^		Restricted controls^**b**^
		**Odds ratio (95% CI)**	*p* value	**Odds ratio (95% CI)**	*p* value	**Odds ratio (95% CI)**
**Intrauterine death**	270/78,318 (0.34%)					
Any iodine supplement use GW 0–20	29,131	0.80 (0.62, 1.03)	0.089	0.78 (0.61, 1.02)	0.071	0.96 (0.64, 1.45)
First report of iodine supplement						
Never (non-supplement user)	49,187 (13,451^b^)	1 (ref.)		1 (ref.)		1 (ref.)
Before pregnancy^c^	7477	0.79 (0.51, 1.23)	0.30	0.76 (0.48 1.19)	0.23	0.92 (0.53, 1.62)
GW 0–12	7149	0.94 (0.62, 1.42)	0.77	0.92 (0.60, 1.40)	0.70	1.11 (0.63, 1.94)
GW > 12	5253	0.77 (0.45, 1.30)	0.32	0.76 (0.45, 1.29)	0.31	0.78 (0.38, 1.60)
**Preeclampsia**	2936/78,318 (3.8%)					
Any iodine supplement use GW 0–20	29,131	0.98 (0.91, 1.06)	0.64	0.93 (0.86, 1.01)	0.098	0.94 (0.84, 1.06)
First report of iodine supplement						
Never (non-supplement user)	49,187 (13,451^b^)	1 (ref.)		1 (ref.)		1 (ref.)
Before pregnancy^c^	7477	0.88 (0.77, 1.01)	0.067	0.85 (0.74, 0.98)	0.022	0.84 (0.71, 1.00)
GW 0–12	7149	1.05 (0.92, 1.19)	0.48	0.96 (0.84, 1.10)	0.58	0.97 (0.82, 1.14)
GW > 12	5253	0.99 (0.86, 1.15)	0.93	0.93 (0.80, 1.08)	0.35	0.91 (0.76, 1.11)
**Preterm delivery (< GW 37)**	3889/77,995 (5.0%)					
Any iodine supplement use GW 0–20	29,014	1.00 (0.93, 1.07)	0.98	0.97 (0.91, 1.04)	0.42	1.07 (0.96, 1.18)
First report of iodine supplement						
Never (non-supplement user)	48,981 (13,405^b^)	1 (ref.)		1 (ref.)		1 (ref.)
Before pregnancy^c^	7445	1.07 (0.96, 1.20)	0.21	1.05 (0.94, 1.18)	0.35	1.18 (1.03, 1.36)
GW 0–12	7126	1.02 (0.91, 1.14)	0.70	0.99 (0.88, 1.11)	0.82	1.08 (0.94, 1.25)
GW > 12	5230	1.02 (0.90, 1.17)	0.71	0.99 (0.87, 1.13)	0.86	1.04 (0.89, 1.23)
**Early preterm delivery (< GW 32)**	612/77,995 (0.78%)					
Any iodine supplement use GW 0–20	29,014	0.82 (0.69, 0.97)	0.020	0.80 (0.67, 0.95)	0.010	0.82 (0.64, 1.05)
First report of iodine supplement						
Never (non-supplement user)	48,981 (13,405^b^)	1 (ref.)		1 (ref.)		1 (ref.)
Before pregnancy^c^	7445	0.94 (0.72,1.24)	0.67	0.94 (0.71, 1.24)	0.66	0.98 (0.71, 1.38)
GW 0–12	7126	0.83 (0.62,1.12)	0.22	0.80 (0.60, 1.08)	0.14	0.73 (0.51, 1.08)
GW > 12	5230	0.61 (0.41, 0.90)	0.014	0.59 (0.40, 0.87)	0.008	0.62 (0.39, 0.98)
**Spontaneous preterm delivery (< GW 37)**	2211/76,313 (2.8%)					
Any iodine supplement use GW 0–20	28,406	1.03 (0.95, 1.13)	0.45	1.00 (0.91, 1.09)	0.93	1.09 (0.95, 1.25)
First report of iodine supplement						
Never (non-supplement user)	47,907 (13,196^b^)	1 (ref.)		1 (ref.)		1 (ref.)
Before pregnancy^c^	7273	1.08 (0.94, 1.25)	0.28	1.07 (0.92, 1.24)	0.39	1.20 (1.00, 1.44)
GW 0–12	6977	1.08 (0.93, 1.25)	0.31	1.02 (0.88, 1.19)	0.75	1.12 (0.93, 1.35)
GW > 12	5123	1.09 (0.93, 1.29)	0.29	1.04 (0.88, 1.23)	0.65	1.12 (0.91, 1.38)
		**Beta (95% CI)**		**Beta (95% CI)**		**Beta (95% CI)**
**Birth weight**	78,210					
Any iodine supplement use GW 0–20	29,091	− 11.0 (− 19.4, − 2.7)	0.009	13.6 (5.6, 21.7)	0.001	3.3 (−8.3, 14.8)
First report of iodine supplement						
Never (non-supplement user)	49,119 (13,435^b^)	0 (ref.)		0 (ref.)		0 (ref.)
Before pregnancy^c^	7462	− 5.1 (− 19.1, 8.9)	0.48	16.3 (2.7, 29.9)	0.019	7.5 (− 9.0, 23.9)
GW 0–12	7139	− 12.4 (− 26.4, 1.6)	0.084	19.6 (6.0, 33.1)	0.005	9.7 (− 6.8, 26.2)
GW > 12	5248	− 11.7 (− 27.7, 4.3)	0.15	16.8 (1.4, 32.2)	0.033	12.0 (− 6.5, 30.4)
**Birth weight*z*-score**	77,949					
Any iodine supplement use GW 0–20	28,998	− 0.03 (− 0.04, − 0.01)	0.001	0.03 (0.01, 0.04)	< 0.001	0.01 (− 0.01, 0.03)
First report of iodine supplement						
Never (non-supplement user)	48,951 (13,393^b^)	0 (ref.)		0 (ref.)		0 (ref.)
Before pregnancy^c^	7439	0.00 (−0.02, 0.03)	0.88	0.05 (0.03, 0.07)	< 0.001	0.04 (0.01, 0.07)
GW 0–12	7121	− 0.03 (− 0.05, − 0.00)	0.043	0.04 (0.02, 0.07)	< 0.001	0.03 (0.00, 0.06)
GW > 12	5229	− 0.04 (− 0.07, − 0.02)	0.002	0.02 (− 0.01, 0.05)	0.13	0.01 (− 0.02, 0.05)
		**Odds ratio (95% CI)**		**Odds ratio (95% CI)**		**Odds ratio (95% CI)**
**SGA**	7637/77,949 (9.8%)					
Any iodine supplement use GW 0–20	28,998	1.01 (0.96, 1.06)	0.83	0.91 (0.87, 0.96)	< 0.001	0.95 (0.88, 1.02)
First report of iodine supplement						
Never (non-supplement user)	48,951 (13,393^b^)	1 (ref.)		1 (ref.)		1 (ref.)
Before pregnancy^c^	7439	0.97 (0.89, 1.06)	0.49	0.89 (0.82, 0.97)	0.009	0.91 (0.82, 1.01)
GW 0–12	7121	0.98 (0.90, 1.07)	0.70	0.88 (0.80, 0.95)	0.003	0.91 (0.82, 1.02)
GW > 12	5229	1.04 (0.94, 1.14)	0.44	0.93 (0.84, 1.02)	0.139	0.95 (0.85, 1.07)
**LGA**	7539/77,949 (9.7%)					
Any iodine supplement use GW 0–20	28,998	0.92 (0.88, 0.97)	0.002	1.03 (0.98, 1.08)	0.31	1.02 (0.95, 1.10)
First report of iodine supplement						
Never (non-supplement user)	48,951 (13,393^b^)	1 (ref.)		1 (ref.)		1 (ref.)
Before pregnancy^c^	7439	1.00 (0.92, 1.08)	0.92	1.10 (1.01, 1.19)	0.034	1.08 (0.97, 1.19)
GW 0–12	7121	0.89 (0.82, 0.97)	0.011	1.03 (0.94, 1.12)	0.57	1.02 (0.92, 1.14)
GW > 12	5229	0.88 (0.80, 0.97)	0.014	1.01 (0.91, 1.12)	0.89	1.01 (0.90, 1.14)

^a^Models were adjusted for maternal age, BMI, parity, education, smoking in pregnancy, fibre intake, chronic illness, in vitro fertilization, folic acid supplement within the interval from 4 weeks before to 8 weeks after conception (only for intrauterine death), child sex (only for unstandardized birth weight), and vitamin D (only for preeclampsia)

^b^Adjusted associations restricting the reference group (non-users) to participants who reported use of one or more multivitamin/multimineral supplements in the food frequency questionnaire, but not any containing iodine

^c^One to 26 weeks before conception

There was no evidence of effect modification by habitual iodine intake from food for any of the associations studied between iodine supplement use and outcomes. Also, supplement use was not associated with iodine intake from food. Thus, models presented were not adjusted for iodine intake from food.

In light of results in previous studies in MoBa [28, 29], we additionally tested a potential confounding effect of use of milk products containing probiotic bacteria (yes/no) in the models with the outcomes preeclampsia and preterm delivery, and for preeclampsia a potential confounding effect of vitamin D supplement use. The results did not change, and therefore, these variables were not included in the final models.

### Child anthropometrics at birth

**Fig. 4:**
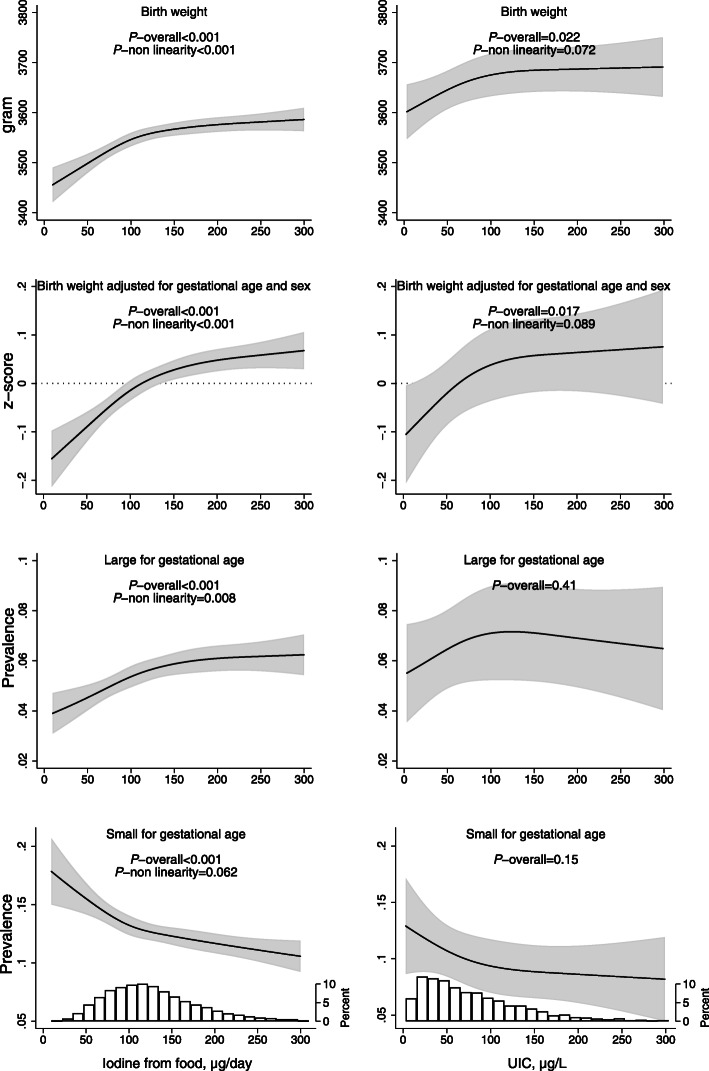
Associations between measures of maternal iodine intake and child birth weight. In the left column, the exposure is iodine from food in non-users of iodine-containing supplement, and in the right column, the exposure is urinary iodine concentration (including supplement users) (*n* = 2795). Associations are adjusted for maternal age, BMI, parity, education, smoking in pregnancy, energy intake, and fibre intake. Sample size: birth weight *n* = 49,119, *z*-score *n* = 48,951 (incl. 9.8% SGA, 9.9% LGA). For the crude associations, Additional file 1: Figure S7

A low iodine intake from food (less than ~ 150 μg/day) as well as a low UIC (below ~ 100 μg/L) was associated with lower birth weight and lower birth weight *z*-score (adjusted for gestational length, child sex and standardized) (Fig. 4). Compared with an intake of 150 μg/day (reference), mean *z*-score was 0.04 SD lower at 100 μg/day (95% CI − 0.06, − 0.02) and 0.08 SD lower at 75 μg/day (95% CI − 0.10, − 0.06) (*p* overall < 0.001). In full-term babies born in GW 40, a 0.08 SD difference in *z*-score corresponds to 36 g. A low iodine intake from food was also associated with a reduced risk of being LGA (i.e. having a birth weight in the top 90 percentile for child sex and gestational age at birth) and an increased risk of being SGA (< 10 percentile) (Fig. 4). Results did not change when restricting the definition of SGA to the below percentiles 5 or 3 on birth weight *z*-score (results not shown). The curve shapes indicated similar associations for UIC, but they did not reach statistical significance (Fig. 4).

Iodine intake from food was also associated with placenta weight and head circumference, but when adjusting for child birth weight, the associations were no longer present (Additional file 1: Figure S6). Again, the curve shapes indicated similar associations for UIC, but they did not reach statistical significance (results not shown).

Use of an iodine-containing supplement was associated with increased birth weight, but the estimates were attenuated when restricting the reference group to participants using nutrient supplements other than the standard recommended ones (Table 3). Then, only long-term use (initiated before pregnancy) remained statistically significant. However, use of iodine-containing supplements was associated with a reduced risk of being SGA (aOR 0.91 (0.87, 0.96), *p* < 0.001), and long-term use was associated with an increased risk of being LGA (aOR 1.10 (1.01, 1.19), *p* = 0.034). Overall, the results indicated that use of iodine supplements increased birth weight and especially long-term use (+ 0.05 SD or + 22 g in full-term babies).

## Discussion

The main finding in this uniquely large pregnancy cohort is that a low iodine intake (lower than ~ 150 μg/day) was consistently associated with reduced foetal growth across all three measures of exposure (i.e. iodine from food, UIC, and iodine supplement use). This strengthens the evidence of a causal relationship. Also, both a low iodine intake from food (lower than ~ 100 μg/day) and no iodine supplement use were associated with an increased risk of preeclampsia. This association was not detected for UIC, but UIC was only measured in a subsample of women, and a single spot UIC provides a very poor measure of iodine status at the individual level. Thus, the analyses with UIC as the measure of exposure were underpowered to detect small differences in risk for dichotomous outcomes. A low iodine intake from food was associated with an increased risk of preterm delivery, but the results for supplement use on this outcome were not consistent. We also found indications that low iodine intake from food was associated with an increased risk of subfecundity, but the design of this study, including only women who had already succeeded in becoming pregnant and measuring the exposure after the outcome, was far from optimal to study the association with this outcome.

Median iodine intake from food was 121 μg/day, and median UIC was 69 μg/L. These figures show that the study sample had insufficient iodine intake according to international recommendations for iodine intake in pregnant as well as in non-pregnant women. Insufficient iodine intake is by WHO defined in pregnant women with a median UIC < 150 μg/L [27], and this corresponds to the recommended iodine intake of 250 μg/day by the WHO [27]. In our study, median UIC in non-supplement users was 59 μg/L and as low as 32 μg/L in non-dairy consumers. In women reporting current supplement use, the median UIC was 99 μg/L. A median UIC in the range 30–74 μg/L has been suggested to define moderate iodine deficiency [15]; thus, our results might reflect the impact of moderate iodine deficiency rather than mild. In fact, we could not identify any subgroup in our population with median UIC > 150 as even long-term supplement users had a median UIC < 150 μg/L. Thus, our study might not include women representative of the optimal range of iodine intake for pregnant women.

### Foetal growth

A low habitual iodine intake (lower than about 150 μg/day) was associated with lower birth weight and a lower birth weight *z*-score (adjusted for gestational age and sex). It was also associated with an increased risk of being SGA and a decreased risk of being LGA and a proportionally lower head circumference and placenta weight. Our results show that use of iodine-containing supplements might increase foetal growth to some extent, indicating a causal association. Severe iodine deficiency has been documented to reduce birth weight and increase the risk of being SGA [7], but only few studies have reported lower birth weight or changes in other measures of foetal growth in mild-to-moderate iodine deficiency [13, 14]. Several studies have reported null findings, including the ALSPAC cohort in the UK (median UIC 95 μg/L, *n* = 3140) [10], the INMA cohort in Spain (median UIC = 128 μg/L, *n* = 1908) [12], and the SCOPE cohort in the UK (median UIC = 134 μg/L, *n* = 541) [11]. A recent meta-analysis including 13 studies and ~ 11,000 newborns found no association between UIC and birth anthropometrics [30]. However, the meta-analysis included mostly populations that were iodine sufficient on a group level (i.e. median UIC > 150 μg/L). All in all, studies to date have major weaknesses and also do not have the power to detect potential minor changes in foetal growth parameters. The studies are generally too small, iodine status is mostly close to sufficient or sufficient at a group level, a single spot UIC is used as a measure of iodine status, and power is often further drastically reduced by categorizing the exposure, and sometimes also the outcome variables, into two or more categories. In some studies, the UIC exposure variable is corrected for hydration by dividing it by urinary creatinine concentration. Although this provides a more accurate measure of iodine status at an individual level, it at the same time introduces bias in the models when investigating growth outcomes since maternal creatinine excretion varies with maternal factors like age, BMI, and fitness level, making the results difficult to interpret [31].

In a systematic review investigating the effect of iodized salt and iodine supplements on prenatal and postnatal growth, Farebrother et al. [15] found that iodine supplementation of severely iodine-deficient women (median UIC < 30 μg/L) increased mean birth weight by 200 g and that iodine repletion in milder deficiencies showed no effect on prenatal growth, but the quality of the evidence was very low [15]. The authors concluded that there were few well-designed trials studying effects of iodine repletion on prenatal growth and that potential effects remain understudied [15].

Milk intake has been reported to be positively associated with birth weight, and the evidence was reported to be limited, but suggestive in a systematic review published in 2012 [32]. Milk contains a number of nutrients and bioactive compounds that potentially can affect foetal growth, but it is also an important dietary source of iodine in the countries of the studies that were included in the systematic review [32]. The very high correlation between iodine intake and reported intake of milk/yoghurt in our study (*r* = 0.85*)* made it impossible to control for milk intake in our analyses. However, the positive effect of iodine supplements on foetal growth strengthens the evidence for a causal link between iodine and foetal growth in this moderately iodine-deficient population.

### Intrauterine death, preeclampsia, and preterm delivery

Abortion, stillbirth, and perinatal mortality are all listed among the consequences of severe iodine deficiency [7]. In our study, we did not find an association between iodine intake and intrauterine death, but the total prevalence of this outcome in MoBa was low (0.34%), and all participants had already reached GW 22 at inclusion in our study sample. Our null finding therefore does not exclude the possibility of mild-to-moderate iodine deficiency being linked to pregnancy loss.

Preeclampsia was more prevalent in women with iodine intakes lower than ~ 100 μg/day, and initiating use of iodine-containing supplements before pregnancy was associated with a decrease in risk (aOR 0.85 (95% CI 0.74, 0.98)). Several risk factors are currently known to be associated with hypertensive disorders in pregnancy, including thyroid function [33]. In a US retrospective cohort study including *n* = 223,512 singleton pregnancies, both hypo- and hyperthyroidism were associated with an increased risk of preeclampsia [34]. Results from the Generation R-study (*n* = 5153) show that also high-normal FT4 levels during early pregnancy were associated with an increased risk of preeclampsia [35]. In MoBa, we have previously reported that a low UIC was associated with a higher FT4 in GW 18 [4]. Only few studies have investigated the association between iodine status and risk of preeclampsia, but they were underpowered to investigate small differences in risk by iodine status and reported null findings [10, 36].

Our results on preterm delivery are less consistent. Although a low iodine intake from food was associated with an increased risk of preterm delivery (*p* overall = 0.003), we found no association with the risk of early preterm delivery. Neither did the results on supplement use show any clear trends on preterm delivery. Thyroid (dys-)function has been linked to the risk of preterm delivery [34, 37], so we cannot exclude the possibility of an increased risk in iodine deficiency. In both ALSPAC [10] and SCOPE [11], there was no association between UIC and preterm delivery, but again, these studies were underpowered to detect small differences in risks.

### Subfecundity

The prevalence of subfecundity is estimated to be one in every seven women of childbearing age in high-income countries and one in four in low-income countries [38]. Thyroid hormones are known to be important in the regulation of reproductive tissues, and thyroid disorders are common and associated with increased risk of subfecundity [1, 39]. To our knowledge, only one study has investigated if iodine deficiency may be linked to fecundity, but this study was performed in a population that was iodine sufficient at a group level (median UIC 113 μg/L) [9]. The results showed an estimated 46% reduction in fecundity (*p* = 0.028) in women with a spot UIC < 50 μg/g creatinine vs. ≥ 100 [9]. We also found that a low iodine intake (lower than ~ 100 μg/day) was associated with an increased risk of subfecundity although our sample only included women who had succeeded in becoming pregnant and remaining pregnant up to inclusion at mid-pregnancy. However, differences in risk were low and might have been attenuated by the study design. Recently, a study was published showing that women with reproductive failures had more iodine transporters in the endometrium (> 5-fold increase in mRNA levels of the iodine transporters NIS and PENDRIN compared to healthy women with at least one successful pregnancy), and the authors suggest the results might indicate suboptimal iodine intake in the women with reproductive failures [40]. In livestock, adding iodine to the feed increases fecundity in areas with iodine deficiency [8]. Therefore, weak evidence suggests that iodine deficiency in humans reduces fecundity, but more studies are needed, and preferably with a prospective design.

### Strengths and limitations

The strengths of this study include its uniquely large sample size (*n* = 78,318), the population-based, prospective design, the extensive data collection, the near-complete follow-up using data from the national birth registry, and the fact that there were three different measures of exposure available (i.e. iodine intake by an extensive and validated FFQ, reported supplement use, and UIC in spot urine samples). Additionally, there was a large variation in exposure between women as a result of few food sources of iodine, and the population had insufficient iodine intake at a group level.

A major limitation of the study was that only few of the participants had a calculated iodine intake above the recommended intake; thus, the study may not include participants with an optimal intake of iodine for comparison. However, recommendations are set with a safety margin to assure an adequate intake at a group level. Also, knowledge on what constitutes an optimal iodine intake in pregnancy is limited. This is reflected in the wide variation in recommendations in different regions of the world ranging from 140 μg/day in the UK [41] to 250 μg/day by WHO [27]. Our results indicate that an adverse impact on the outcomes studied was seen at an intake below ~ 100–150 μg/day and that the association curves plateaued at higher intake. This range is well within the intake range of our study population.

The observational design means that we cannot rule out the possibility of residual confounding. However, iodine intake measured by all three exposures was fairy equal across maternal background factors including socioeconomic factors, age, BMI, and fibre as a proxy for a healthy diet, and this probably reduces the risk of residual confounding. Also, the pregnant women in MoBa were generally well nourished [25] making confounding by other nutrient deficiencies less likely. Unfortunately, the strong correlation between iodine intake and milk/yoghurt intake made it impossible to control for milk intake in the analyses in this study. Thus, we cannot exclude the possibility that other nutrients or bioactive compounds in milk may have confounded the associations. This would affect both the analyses with calculated iodine intake from food and UIC, but not with iodine supplements.

UIC was only available for a subsample of women (*n* = 2795), and the analyses using UIC as exposure was underpowered to detect small changes in dichotomous outcomes due to the large measurement error in iodine status when using UIC as a proxy [42]. The data on supplement use (frequency and dosage) was also limited so we could only investigate “any use” vs. “no use” in different time periods, and not dose-response. The low UIC in participants who had reported taking iodine-containing supplements in pregnancy (UIC = 85 μg/L) indicated that many women did not consume such supplements over time and on a regular basis. This might have attenuated the results on the impact of supplement use.

Although we had three different measures of exposure, none of them can be considered a very good proxy for maternal iodine status. Considerable measurement error in measuring food intake and urinary excretion would contribute to weaken the associations with outcomes.

Selection bias has most likely contributed to attenuate the results for the outcomes subfecundity, intrauterine death, and preterm delivery since our study sample only included women who actually succeeded in getting pregnant and remained pregnant up to inclusion in our study sample at GW 22 (food frequency questionnaire).

### Clinical relevance and implications

Mild-to-moderate iodine deficiency is highly prevalent both in low- and high-income countries, and especially in pregnant women [43–45]. Although the WHO recommends salt iodization to prevent iodine deficiency, many countries have still not implemented adequate measures. Due to changes in food consumption patterns characterized by decreases in milk consumption and a low fish intake, iodine deficiency is currently re-emerging in countries that were previously defined as iodine sufficient [44, 46, 47]. Thus, even marginal changes in risks or in foetal growth might be clinically relevant at a population level. Preterm delivery and reduced foetal growth are associated with neonatal morbidity and mortality and have huge public health implications for the society as well as the families involved [48–51]. Likewise, preeclampsia is additionally associated with maternal mortality and morbidity [52]. The prevalence of preterm delivery is about 11% worldwide [53], foetal growth restriction affects around 10% of all pregnancies [50], and preeclampsia affects around 5% of all pregnancies [52].

Use of iodine supplements may help meet the increased iodine needs during pregnancy. WHO recommends iodine supplements for pregnant women in areas of inadequate iodine intake [54]. However, there is insufficient data from randomized controlled trials to draw meaningful conclusions on the benefits and harms of routine iodine supplementation in all pregnant women [55]. In women with severe iodine deficiency, iodine supplementation reduces the risk of thyroid hypofunction, while in women who are mild-to-moderately iodine deficient, studies are not consistent [56]. Some studies indicate that initiating iodine supplementation in early pregnancy may result in a temporary “stunning effect” of the thyroid resulting in lower thyroid hormone production [57, 58]. Other studies report that iodine supplement use may be beneficial or have no effect [55, 59]. The results from our study indicate that iodine status needs to be corrected by supplement use and/or food iodization before pregnancy to protect the foetus from iodine deficiency.

We suggest that the results in our study should be used for power calculation when planning future studies on adverse pregnancy outcomes to secure an adequately large sample size and that the relevant range of iodine intakes is studied (i.e. median UIC < 100–150 μg/L). Since spot UIC provides only very limited information about iodine intake at the individual level, a simplified iodine nutrition survey could provide a better indicator in countries where iodized salt is not an important dietary source of iodine. When studying foetal growth as an outcome, both exposure and outcome should be on a continuous scale to increase the chance of detecting small changes.

## Conclusions

Insufficient iodine intake was associated with reduced foetal growth and increased risk of preeclampsia in this mild-to-moderately iodine-deficient pregnant population. There were also indications of increased risk of preterm delivery and subfecundity. The results indicate that ideally, iodine deficiency should be prevented in *all* women of fertile age and that initiating iodine supplement use in pregnancy may be too late.

## Supplementary information

**Additional file 1: Figure S1.** Simplified directed acyclic graph (DAG) illustrating the association between maternal iodine intake in pregnancy and pregnancy outcomes. **Table S1**. Adjusted associations between iodine intake from food in pregnancy in non-users of iodine-containing supplements and pregnancy outcomes. **Table S2.** Adjusted associations between iodine intake from food in pregnancy in non-users of iodine-containing supplements and birth anthropometrics1 **Table S3.** Adjusted associations between urinary iodine concentration (GW18) and birth anthropometrics. **Table S4.** Characteristics of the study population by exposure (*n* = 78,318). **Figure S2**. Crude association between habitual iodine intake from food (GW 0–22) and estimated prevalence of subfecundity (> 12 months trying to get pregnant) in planned pregnancies. **Figure S3**. Iodine from food and prevalence of adverse pregnancy outcomes in non-users of iodine-containing supplement, crude and adjusted models. **Figure S4**. Associations between maternal habitual iodine intake and prevalence of iatrogenic and spontaneous preterm delivery (GW < 37) in non-users of iodine-containing supplements. **Figure S5.** Iodine intake from food or UIC and prevalence of preeclampsia and preterm delivery (<GW37) in non-users of iodine-containing supplements. **Figure S6.** Associations between maternal habitual iodine intake and child head circumference and placenta weight in non-users of iodine-containing supplements. **Figure S7.** Associations between measures of maternal iodine intake and child birth weight - crude models.

## Data Availability

The data that support the findings of this study are available from the Norwegian Institute of Public Health https://www.fhi.no/en/studies/moba/for-forskere-artikler/research-and-data-access/ but restrictions apply to the availability of these data, which were used under license for the current study, and so are not publicly available. Data are however available from the authors upon reasonable request and with permission of the Norwegian Institute of Public Health.

## Abbreviations

aOR: Adjusted odds ratio
BMI: Body mass index
CI: Confidence interval
DAG: Directed acyclic graph
FFQ: Food frequency questionnaire
FT4: Free thyroxine
GW: Gestational week
LGA: Large for gestational age
IQR: Interquartile range
MBRN: Medical Birth Registry of Norway
MoBa: Norwegian Mother, Father and Child Cohort Study
SD: Standard deviation
SGA: Small for gestational age
T3: Triiodothyronine
T4: Thyroxine
UIC: Urinary iodine concentration
WHO: World Health Organization
