# Supplementary material for: Insufficient maternal iodine intake is associated with subfecundity, reduced foetal growth, and adverse pregnancy outcomes in the Norwegian Mother, Father and Child Cohort Study
Source: BMC Med. 2020 Aug 11;18:211. doi: 10.1186/s12916-020-01676-w (PMC7418397; doi:10.1186/s12916-020-01676-w)
Supplement: Supplementary file 1 — Additional file 1: Figure S1. Simplified directed acyclic graph (DAG) illustrating the association between maternal iodine intake in pregnancy and pregnancy outcomes. Table S1. Adjusted associations between iodine intake from food in pregnancy in non-users of iodine-containing supplements and pregnancy outcomes. Table S2. Adjusted associations between iodine intake from food in pregnancy in non-users of iodine-containing supplements and birth anthropometrics1 Table S3. Adjusted associations between urinary iodine concentration (GW18) and birth anthropometrics. Table S4. Characteristics of the study population by exposure (n = 78,318). Figure S2. Crude association between habitual iodine intake from food (GW 0–22) and estimated prevalence of subfecundity (> 12 months trying to get pregnant) in planned pregnancies. Figure S3. Iodine from food and prevalence of adverse pregnancy outcomes in non-users of iodine-containing supplement, crude and adjusted models. Figure S4. Associations between maternal habitual iodine intake and prevalence of iatrogenic and spontaneous preterm delivery (GW < 37) in non-users of iodine-containing supplements. Figure S5. Iodine intake from food or UIC and prevalence of preeclampsia and preterm delivery (<GW37) in non-users of iodine-containing supplements. Figure S6. Associations between maternal habitual iodine intake and child head circumference and placenta weight in non-users of iodine-containing supplements. Figure S7. Associations between measures of maternal iodine intake and child birth weight - crude models. [file 12916_2020_1676_MOESM1_ESM.pdf]

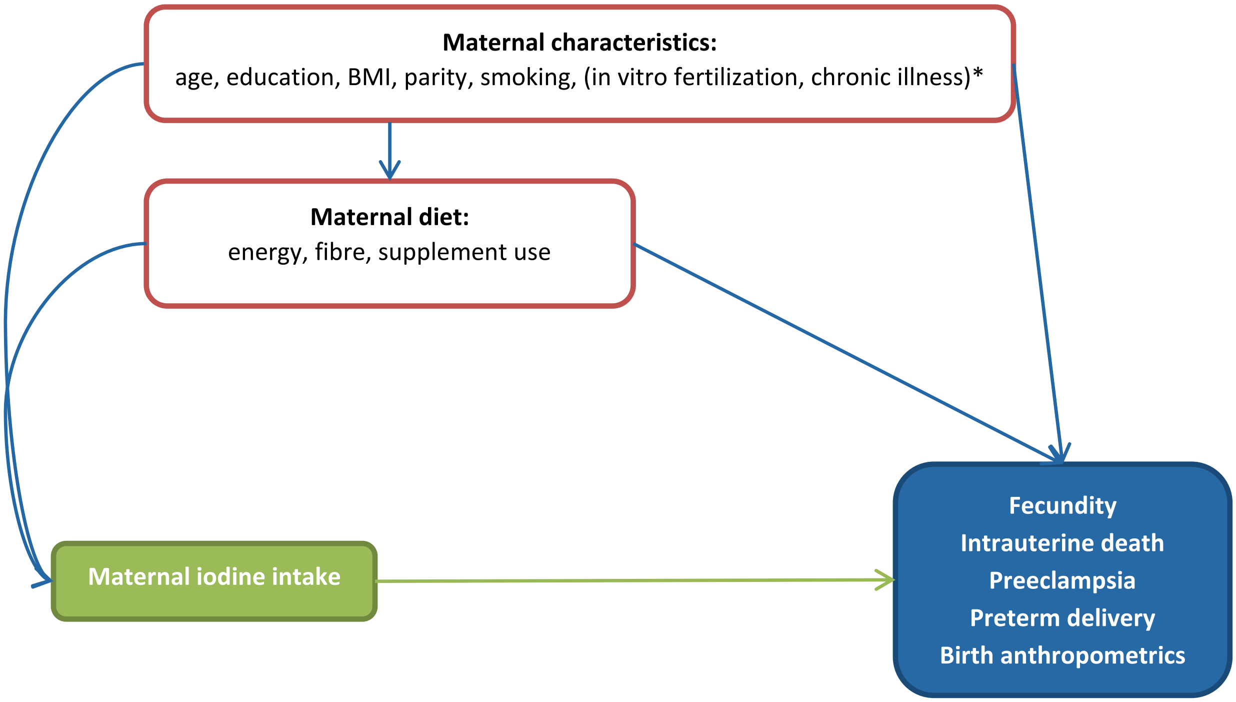

Figure S1 Simplified directed acyclic graph (DAG) illustrating the association between maternal iodine intake in pregnancy and pregnancy outcomes. Confounding factors are illustrated in red boxes. Maternal fibre intake was included as a proxy for diet quality. The green line illustrates the causal pathway. In this paper, we estimate the total effects of iodine on the outcomes, and mediating factors (like thyroid function parameters) are not included in the models.

* In vitro fertilization and chronic illness was included in models when iodine supplement use was the exposure variable since supplement use was more prevalent in IVF-pregnancies and in women with reported chronic illness.

Table S1 Adjusted associations between iodine intake from food in pregnancy in non-users of iodine-containing supplements and pregnancy outcomes[1]

	Subfecundity	Intrauterine death	Preeclampsia	Preterm delivery <GW37	Preterm delivery <GW32	Spontaneous PTD <GW37	Iatrogenic PTD GW<37
**N**	56,416	49,187	49,187	48,981	48,981	47,907[2]	47,575[3]
**n with outcome (%)**	6078 (10.8)	183 (0.35)	1856 (3.8)	2443 (5.0)	412 (0.84)	1371 (2.9)	1039 (2.2)
**Iodine from food, µg/d:**	**Adj OR (95% CI)**	**Adj OR (95% CI)**	**Adj OR (95% CI)**	**Adj OR (95% CI)**	**Adj OR (95% CI)**	**Adj OR (95% CI)**	**Adj OR (95% CI)**
**25**	1.25 (1.07, 1.46)	1.01 (0.63, 1.61)	1.74 (1.35, 2.25)	1.49 (1.18, 1.87)	0.92 (0.54, 1.56)	1.28 (0.94, 1.75)	1.74 (1.25, 2.41)
**50**	1.14 (1.04, 1.26)	1.01 (0.74, 1.37)	1.41 (1.20, 1.64)	1.28 (1.11, 1.47)	0.95 (0.69, 1.32)	1.16 (0.96, 1.41)	1.40 (1.15, 1.72)
**75**	1.05 (1.01, 1.09)	1.00 (0.87, 1.16)	1.14 (1.08, 1.22)	1.10 (1.04, 1.16)	0.99 (0.87, 1.12)	1.06 (0.98, 1.14)	1.15 (1.06, 1.24)
**100 (ref)**	1	1	1	1	1	1	1
**125**	1.02 (0.99, 1.05)	0.99 (0.89, 1.10)	0.99 (0.94, 1.04)	0.99 (0.95, 1.04)	0.96 (0.87, 1.06)	1.00 (0.95, 1.06)	0.98 (0.92, 1.05)
**150**	1.06 (1.00, 1.13)	0.97 (0.81, 1.15)	1.04 (0.93, 1.16)	1.02 (0.93, 1.12)	0.91 (0.73, 1.13)	1.03 (0.92, 1.16)	1.01 (0.87, 1.16)
**175**	1.10 (1.02, 1.19)	0.94 (0.75, 1.18)	1.06 (0.92, 1.22)	1.03 (0.91, 1.16)	0.86 (0.65, 1.14)	1.05 (0.90, 1.22)	1.00 (0.83, 1.20)
**200**	1.13 (1.03, 1.23)	0.91 (0.67, 1.23)	1.06 (0.91, 1.23)	1.01 (0.89, 1.15)	0.82 (0.60, 1.11)	1.05 (0.89, 1.24)	0.96 (0.78, 1.17)
**225**	1.14 (1.04, 1.25)	0.88 (0.59, 1.30)	1.04 (0.89, 1.21)	0.99 (0.87, 1.13)	0.78 (0.56, 1.07)	1.05 (0.88, 1.25)	0.90 (0.73, 1.11)
**250**	1.16 (1.05, 1.28)	0.85 (0.52, 1.39)	1.01 (0.85, 1.20)	0.96 (0.83, 1.11)	0.74 (0.53, 1.05)	1.04 (0.87, 1.26)	0.84 (0.67, 1.05)
**300**	1.18 (1.04, 1.35)	0.79 (0.39, 1.60)	0.96 (0.76, 1.21)	0.90 (0.74, 1.09)	0.68 (0.43, 1.05)	1.03 (0.81, 1.32)	0.72 (0.54, 0.98)
***p*-overall**	p=0.005	p =0.80	p<0.001	p=0.003	p =0.38	p =0.49	p<0.001
***p*-non linearity**	p=0.004		p<0.001	p =0.008			p =0.017

[1] The associations was modelled by multivariable regression adjusting for maternal age, BMI, parity, education, smoking, energy intake, and fibre intake.
[2] Iatrogenic preterm deliveries were excluded from the study sample
[3] Spontaneous preterm deliveries were excluded from the study sample

Abbreviations: GW, gestational week; PTD, preterm delivery

Table S2 Adjusted associations between iodine intake from food in pregnancy in non-users of iodine-containing supplements and birth anthropometrics[1]

	Birthweight gram	Birthweight z-score[2]	SGA	LGA	Head circumference cm	Placenta weight gram
**N**	49,119	48,951	48,951	48,951	48,115	47,886
**n with outcome (%)**			4787 (9.8)	4861 (9.9)		
**Iodine from food, μg/d:**	**Adj beta (95% CI)**	**Adj beta (95% CI)**	**Adj OR (95% CI)**	**Adj OR (95% CI)**	**Adj beta (95% CI)**	**Adj beta (95% CI)**
**25**	-94 (-123, -67)	-0.16 (-0.21, -0.11)	1.45 (1.24, 1.70)	0.69 (0.58, 0.82)	-0.21 (-0.30, -0.13)	-23 (-30, -15)
**50**	-68 (-87, -50)	-0.12 (-0.15, -0.09)	1.31 (1.18, 1.45)	0.76 (0.68, 0.85)	-0.16 (-0.22, -0.10)	-16 (-21, -11)
**75**	-42 (-55, -30)	-0.08 (-0.10, -0.06)	1.18 (1.10, 1.27)	0.84 (0.78, 0.90)	-0.11 (-0.14, -0.07)	-9 (-13, -6)
**100**	-21 (-32, -10)	-0.04 (-0.06, -0.02)	1.09 (1.02, 1.17)	0.91 (0.85, 0.97)	-0.06 (-0.09, -0.02)	-4 (-7, -1)
**125**	-8 (-14, -1)	-0.02 (-0.03, -0.01)	1.03 (0.99, 1.07)	0.96 (0.93, 1.00)	-0.02 (-0.04, 0.00)	-1 (-1, 0)
**150 (ref)**	0	0	1	1	0	0
**175**	5 (2, 9)	0.01 (0.01, 0.02)	0.97 (0.95, 0.99)	1.02 (1.00, 1.05)	0.01 (0.00, 0.02)	1 (0, 2)
**200**	9 (3, 15)	0.02 (0.01, 0.03)	0.94 (0.91, 0.98)	1.04 (1.00, 1.08)	0.01 (-0.01, 0.03)	1 (-1, 3)
**225**	12 (2, 22)	0.03 (0.01, 0.04)	0.92 (0.86, 0.97)	1.05 (0.99, 1.11)	0.01 (-0.02, 0.04)	1 (-1, 4)
**250**	14 (1, 28)	0.03 (0.01, 0.05)	0.89 (0.82, 0.97)	1.05 (0.97, 1.14)	0.00 (-0.04, 0.04)	1 (-3, 5)
**300**	16 (-4, 42)	0.04 (0.00, 0.08)	0.84 (0.73, 0.97)	1.07 (0.93, 1.22)	0.01 (-0.08, 0.05)	1 (-5, 8)
***p*-overall**	$p<0.001$	$p<0.001$	$p<0.001$	$p<0.001$	$p<0.001$	$p<0.001$
***p*-non linearity**	$p<0.001$	$p<0.001$	$p=0.062$	$p=0.008$	$p<0.001$	$p<0.001$

[1] The associations was modelled by multivariable regression adjusting for maternal age, BMI, parity, education, smoking, energy intake, and fibre intake. Continuous outcomes were additionally adjusted for child sex.
[2] Birth weight adjusted for gestational age and child sex and standardized

Abbreviations: SGA, small for gestational age (z-score<10th percentile for birth weight); LGA, large for gestational age (z-score>90th percentile for birth weight)

Table S3 Adjusted associations between urinary iodine concentration (GW18) and birth anthropometrics[1]

	Birthweight gram	Birthweight z-score[2]	SGA	LGA	Head circumference cm	Placenta weight gram
**N**	2795	2785	2785	2785	2761	2732
**n with outcome (%)**			229 (8.2)	296 (10.6)		
**Iodine from food, µg/d:**	**Adj beta (95% CI)**	**Adj beta (95% CI)**	**Adj OR (95% CI)**	**Adj OR (95% CI)**	**Adj beta (95% CI)**	**Adj beta (95% CI)**
**25**	-53 (-94, -11)	-0.10 (-0.18, -0.02)	1.30 (0.95, 1.78)	0.83 (0.62, 1.10)	-0.04 (-0.17, 0.08)	-10 (-23, 3)
**50**	-30 (-53, -7)	-0.06 (-0.10, -0.02)	1.16 (0.98, 1.38)	0.90 (0.77, 1.05)	-0.03 (-0.10, 0.04)	-6 (-13, 1)
**75**	-12 (-20, -3)	-0.02 (-0.04, -0.01)	1.06 (1.00, 1.13)	0.97 (0.91, 1.02)	-0.01 (-0.04, 0.01)	-2 (-5, 0)
**100 (ref)**	0	0	1	1	0	0
**125**	6 (1, 12)	0.01 (0.00, 0.02)	0.96 (0.92, 1.01)	1.01 (0.97, 1.05)	0.01 (0.00, 0.03)	2 (0, 3)
**150**	9 (-2, 20)	0.02 (0.00, 0.04)	0.94 (0.86, 1.04)	1.00 (0.93, 1.08)	0.03 (-0.01, 0.06)	3 (-1, 7)
**175**	11 (-7, 28)	0.02 (-0.02, 0.06)	0.93 (0.79, 1.09)	0.99 (0.88, 1.11)	0.04 (-0.02, 0.10)	3 (-3, 10)
**200**	12 (-13, 36)	0.03 (-0.03, 0.08)	0.92 (0.73, 1.14)	0.97 (0.82, 1.14)	0.06 (-0.03, 0.14)	4 (-5, 14)
**225**	13 (-20, 46)	0.03 (-0.04, 0.10)	0.90 (0.67, 1.21)	0.95 (0.77, 1.18)	0.07 (-0.04, 0.18)	5 (-8, 18)
**250**	14 (-26, 54)	0.03 (-0.05, 0.12)	0.89 (0.62, 1.27)	0.94 (0.72, 1.22)	0.09 (-0.05, 0.22)	6 (-10, 21)
**300**	16 (-38, 69)	0.04 (-0.08, 0.15)	0.87 (0.54, 1.40)	0.91 (0.64, 1.29)	0.11 (-0.07, 0.29)	7 (-14, 27)
***p*-overall**	*p*=0.022	*p*=0.017	*p*=0.15	*p*=0.41	*p*=0.26	*p*=0.15
***p*-non linearity**	*p*=0.072	*p*=0.089				

[1] The associations was modelled by multivariable regression adjusting for maternal age, BMI, parity, education, smoking, and fibre intake. Continuous outcomes were additionally adjusted for child sex.
[2] Birth weight adjusted for gestational age and child sex and standardized

Abbreviations: SGA, small for gestational age (z-score<10th percentile for birth weight); LGA, large for gestational age (z-score>90th percentile for birth weight)

Table S4 Characteristics of the study population by exposure (n=78,318)

	All	Iodine from food (µg/day)				Iodine supplement GW 0-22	
		<50	50-99	100-150 µg/day	>150 µg/day	No	Yes
Study sample, n (%)	78,318 (100)	2987 (3.8)	23,072 (29)	28,192 (36)	24,067 (31)	49,187 (63)	29,131 (37)
Maternal age at delivery, mean (SD), years	30.2 (4.5)	29.5 (4.7)	30.2 (4.5)	30.4 (4.4)	30.0 (4.6)	30.2 (4.6)	30.2 (4.5)
Pre-pregnancy BMI, mean (SD), kg/m^2	24.0 (4.3)	24.8 (4.8)	24.1 (4.3)	23.9 (4.2)	24.0 (4.3)	24.1 (4.3)	23.9 (4.2)
Parity, %							
0	47	51	50	46	46	44	54
1	36	35	35	36	35	37	33
2 or more	17	14	15	17	19	19	14
Maternal education, %							
≤12 y	31	41	30	28	33	32	27
13-16 y	43	40	43	44	43	42	44
>16 y	27	19	28	28	24	25	28
Married/cohabitant, %	96.7	95.9	96.8	96.9	96.4	96.7	96.7
Smoking in pregnancy, %							
No	79	73	78	80	78	78	79
Occasionally	16	17	16	15	15	15	15
Daily	5.0	9.4	5.6	5.3	6.7	6.5	5.1
Chronic illness, %	10	14	11	10	10	10	11
Couples income							
Low	26	29	25	25	29	27	25
Medium	41	41	41	41	42	41	41
High	30	26	32	32	26	29	32
Missing	2.7	3.2	2.3	2.5	3.2	2.8	2.4
Iodine from food, median (IQR), µg/day	121 (89, 161)	42 (36, 47)	80 (68, 90)	123 (112, 135)	187 (166, 223)	122 (89, 162)	121 (89, 161)
Iodine supplement in GW 0-22 (%)	37	38	38	37	37	0	100
UIC, median (IQR), µg/L[1]	69 (35, 116)	50 (22, 90)	58 (31, 106)	70 (37, 115)	78 (43, 129)	59 (32, 100)	85 (45, 140)
UIC, median (IQR), µg/g creatinine[1]	91 (61, 139)	53 (40, 96)	78 (51, 123)	92 (64, 143)	105 (74, 154)	80 (54, 115)	113 (75, 183)
Folic acid before/early pregnancy (%)	73	70	74	74	71	67	83
Maternal energy intake, mean (sd), MJ	9.7 (2.6)	7.1 (1.8)	8.4 (1.9)	9.6 (2.1)	11.5 (2.6)	9.7 (2.6)	9.7 (2.6)
Gestational age, mean (SD), weeks	39.5 (2.0)	39.4 (1.9)	39.4 (2.0)	39.5 (2.0)	39.4 (2.0)	39.4 (2.0)	39.5 (1.9)
Birthweight, mean (SD), gram	3591 (567)	3528 (558)	3564 (566)	3598 (563)	3617 (572)	3596 (574)	3584 (556)

[1] Urinary iodine concentration (UIC) was measured in a subsample of n=2795 pregnant women in mean gestational week 18.5 (SD: 1.3).

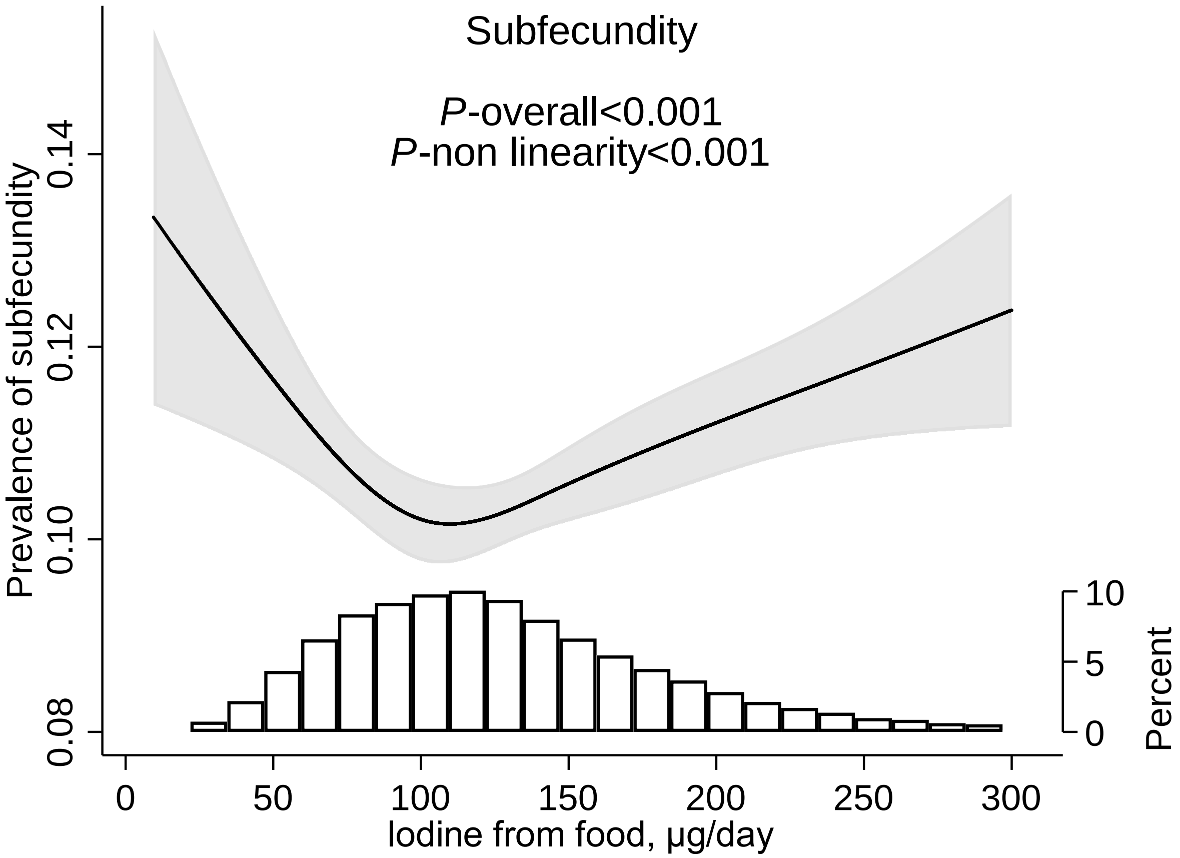

Figure S2 Crude association between habitual iodine intake from food (GW 0-22) and estimated prevalence of subfecundity (>12 months trying to get pregnant) in planned pregnancies (n=56,416, 10.8% subfecundity). The association was modelled by logistic regression adjusting for energy intake. The curve represents the estimated prevalence at mean energy intake, and the shaded area illustrates the 95% robust confidence interval. The histogram shows the distribution of iodine intake from food by the food frequency questionnaire.

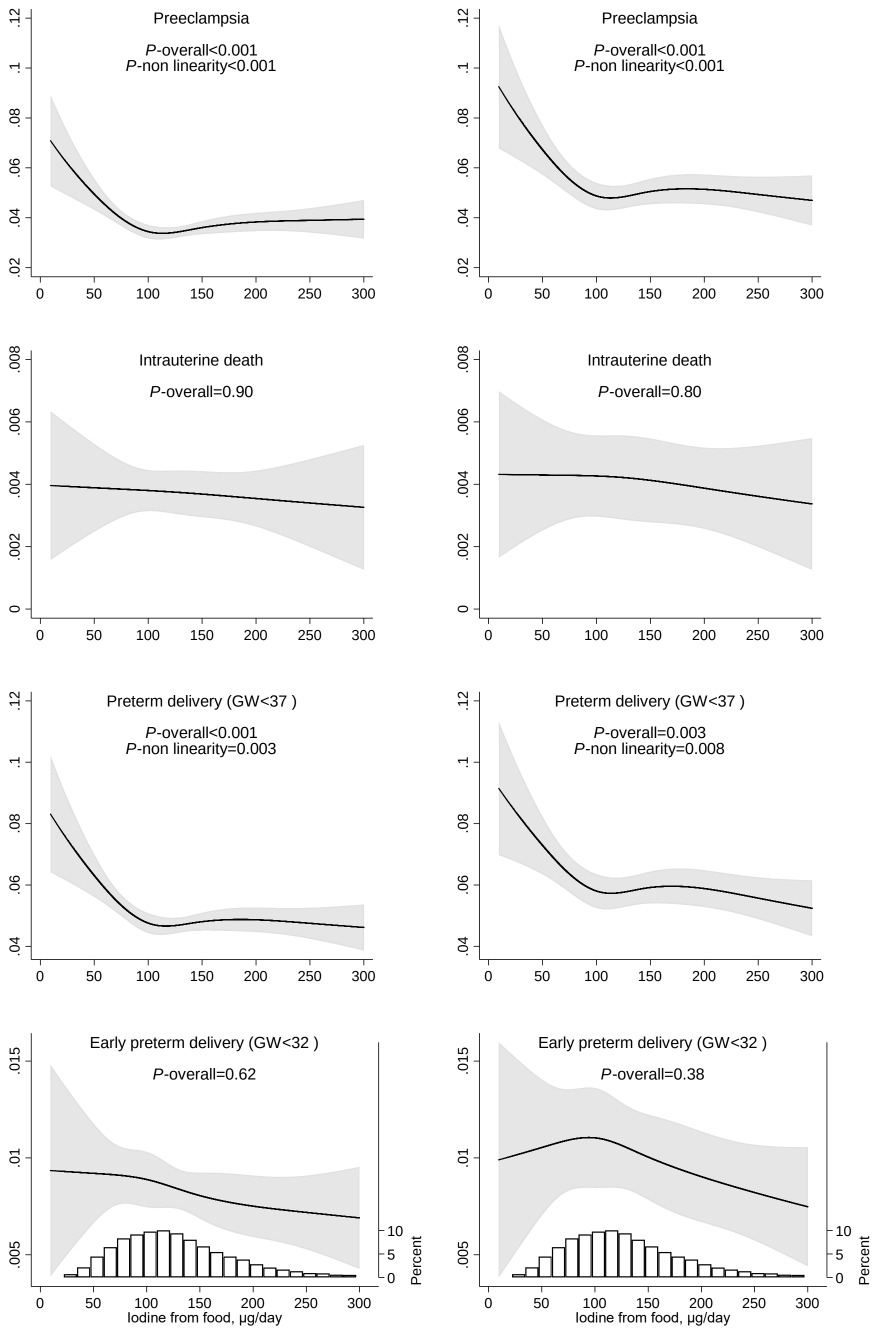

Figure S3 Iodine from food and prevalence of adverse pregnancy outcomes in non-users of iodine-containing supplement. **Crude models are shown in the left column and adjusted models in the right.** Intrauterine death (n=49,187, 0.35% intrauterine deaths), preeclampsia (n=49,187, 3.8% preeclamptic pregnancies) and preterm delivery (n=48,981, 5.0% preterm GW<37 and 0.84% GW<32). Crude models were adjusted for energy intake. Adjusted models were additionally adjusted for maternal age, BMI, parity, education, smoking in pregnancy, and fiber intake. The curve represents the estimated prevalence when covariates were set to their mean, and the shaded area illustrates the 95% robust confidence interval. The histogram shows the distribution of iodine intake from food by the food frequency questionnaire.

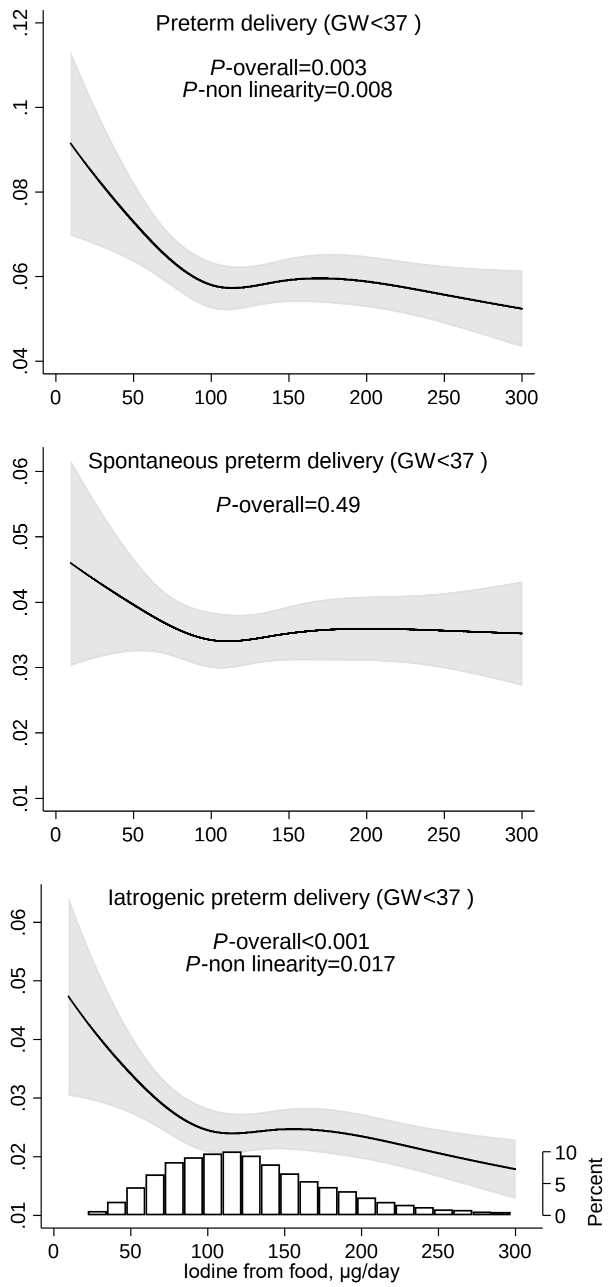

Figure S4 Associations between maternal habitual iodine intake and prevalence of preterm delivery (PTD) (i.e. born before gestational week 37) in non-users of iodine-containing supplements. Sample size for PTD: n=48.981 (5.0% preterm), spontaneous PTD: n=47.907 (2.9% spontaneous) (iatrogenic were excluded), iatrogenic PTD: n=47.575 (2.2% iatrogenic) (spontaneous were excluded). Models were adjusted for maternal age, BMI, parity, education, smoking in pregnancy, energy intake, and fiber intake. The curves represent the estimated prevalence (covariates set to their mean), and the shaded areas illustrate the 95% robust confidence interval. The histogram show the distribution of the exposure.

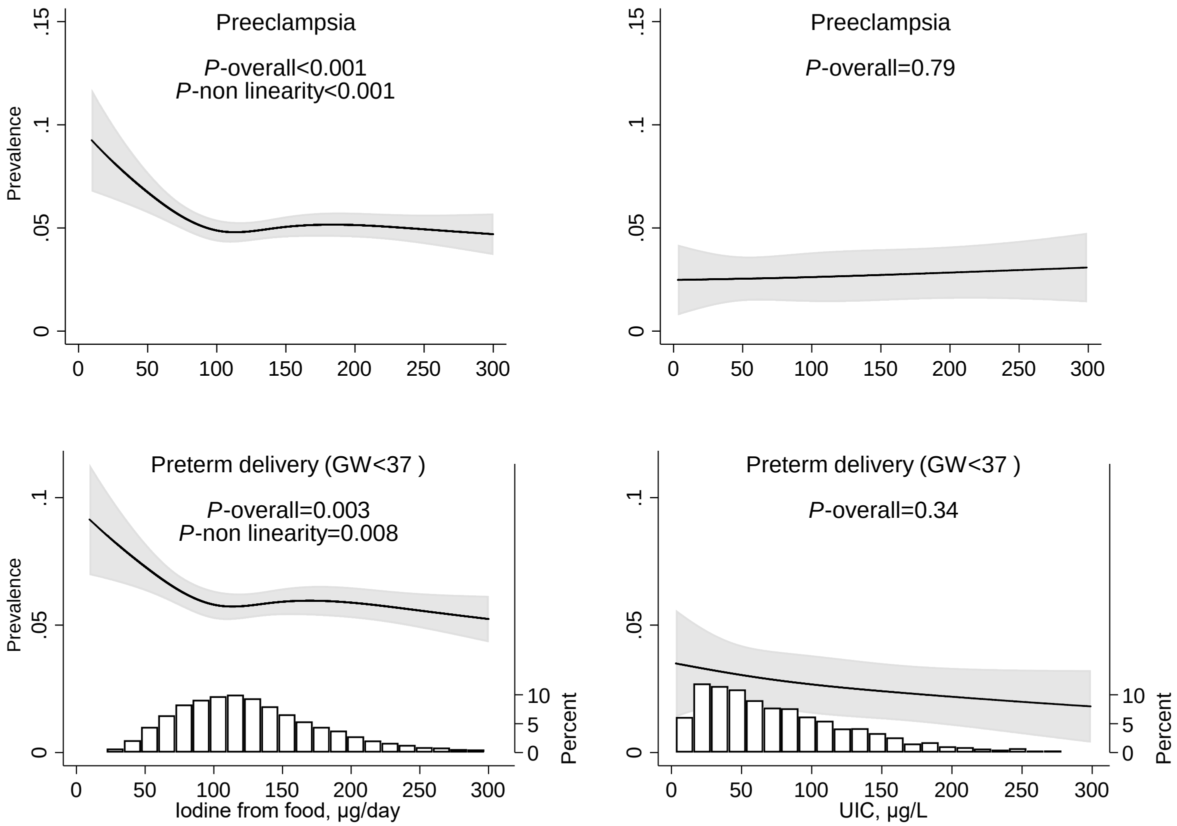

Figure S5

Left column: Iodine intake from food and prevalence of preeclampsia and preterm delivery (<GW37) in non-users of iodine-containing supplements. Sample size was n=49,187 for preeclampsia (3.8% preeclamptic pregnancies) and n=48,981 for preterm delivery (5.0% preterm). Models were adjusted for maternal age, BMI, parity, education, smoking in pregnancy, energy intake, and fiber intake.

Right column: Urinary iodine concentration (UIC) and prevalence of preeclampsia and preterm delivery (<GW37) in a subsample of n=2795 women (including supplement users). Preeclampsia was diagnosed in 2.7% of pregnancies and preterm delivery (<GW37) in 2.9%. Models were adjusted for maternal age, BMI, parity, education, smoking in pregnancy, and fiber intake.

The curves represent the estimated prevalence (covariates set to their mean), and the shaded areas illustrate the 95% robust confidence interval. The histograms show the distribution of the exposures.

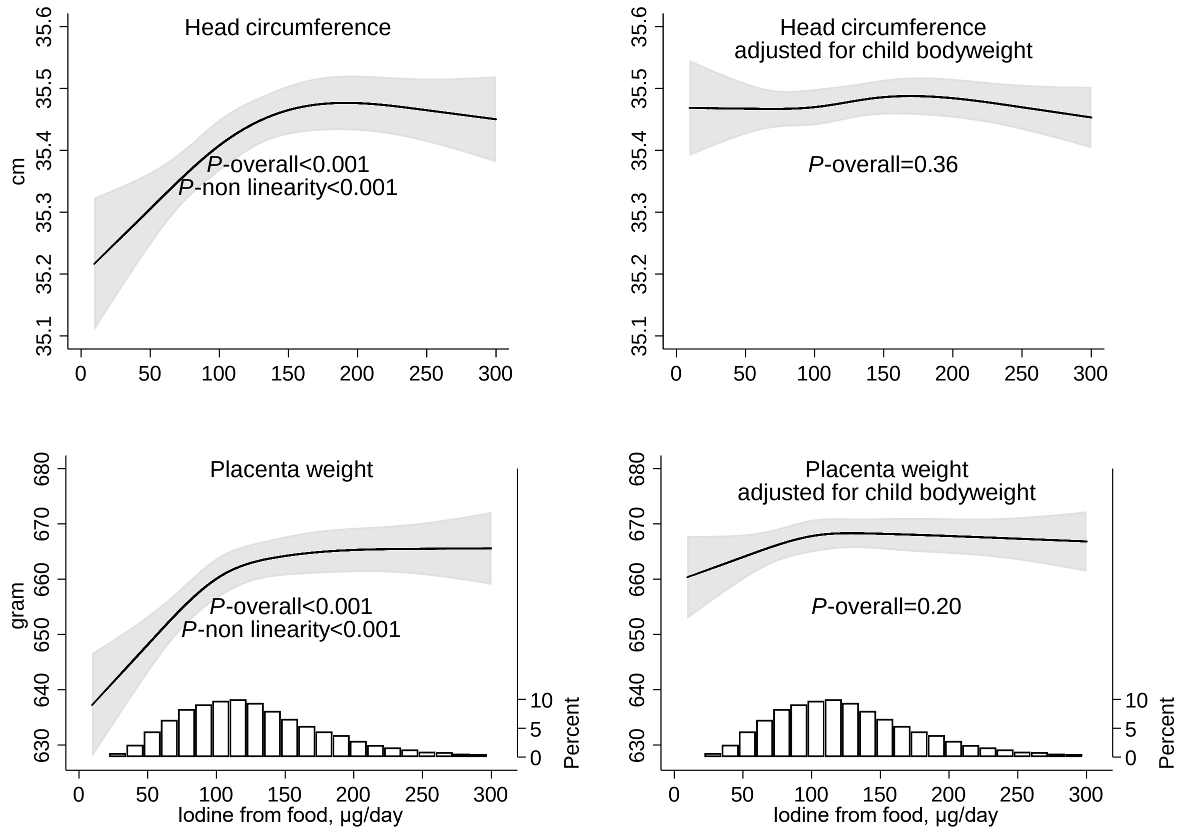

Figure S6 Associations between maternal habitual iodine intake and child head circumference (cm) and placenta weight (gram) in non-users of iodine-containing supplements. In the left column, the models are adjusted for maternal age, BMI, parity, education, smoking in pregnancy, maternal energy intake, maternal and fiber intake. In the right column the models are additionally adjusted for child birthweight. Sample size for head circumference was n=48,150, and for placenta weight: n=47,886. The curve represents the estimated association, and the shaded area illustrates the 95% robust confidence interval. The histogram shows the distribution of the exposure.

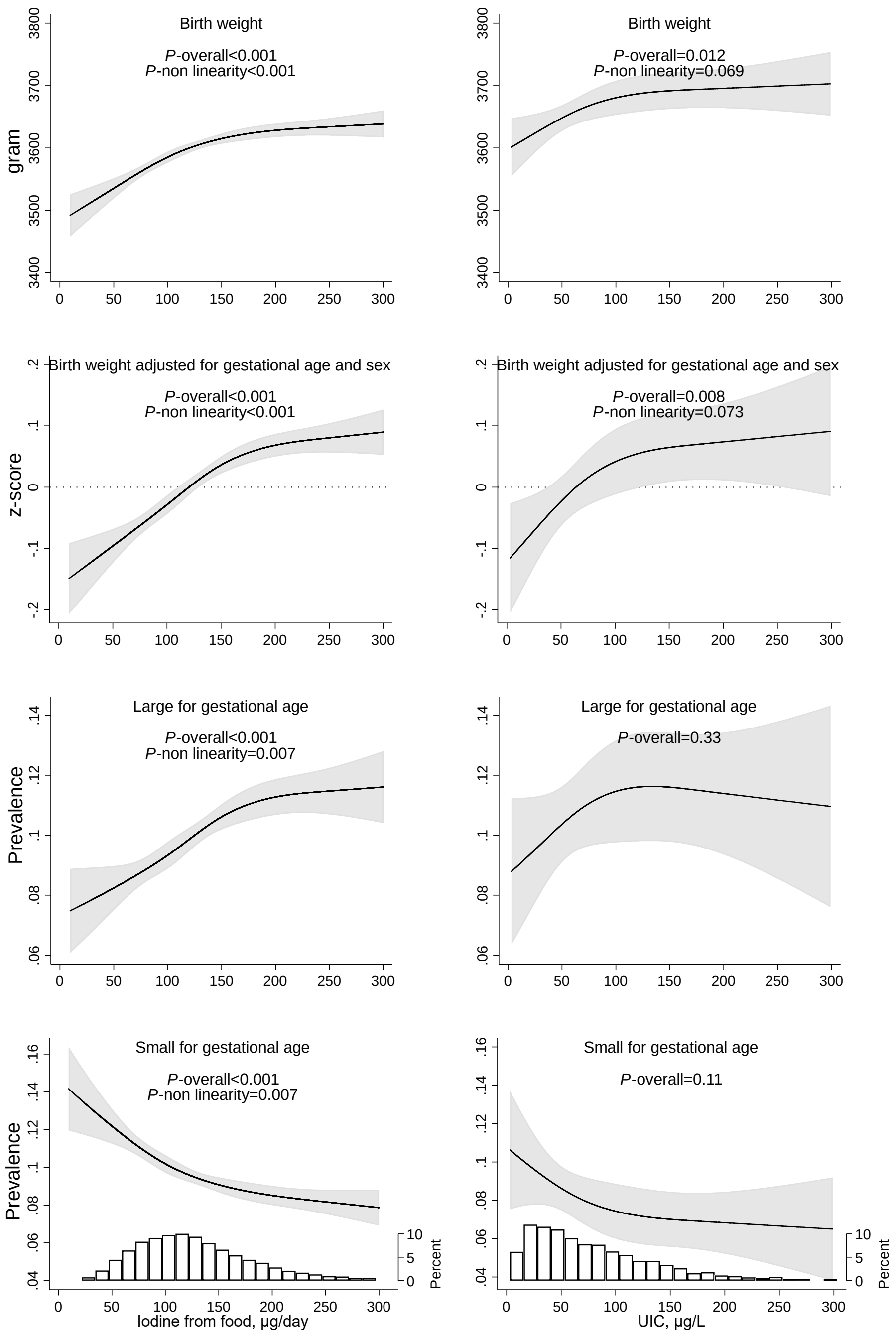

Figure S7 Associations between measures of maternal iodine intake and child birth weight. **Crude models.** In the left column the exposure is iodine intake from food in non-users of iodine-containing supplements, and in the right column the exposure is urinary iodine concentration (including supplement users). Models in the left column are adjusted for energy intake. Sample size left column: birthweight n=49,119, z-score incl. SGA (9.8%) and LGA (9.9%): n=48,951. Sample size right column: n=2795 (8.2% SGA, 10.6% LGA). The curve represents the estimated associations, and the shaded area illustrates the 95% robust confidence interval. The histograms show the distribution of the exposures.
